# Comparative histopathological and immunological study of two field strains of chicken anemia virus

**DOI:** 10.1186/s13567-014-0102-y

**Published:** 2014-10-08

**Authors:** Agustina Rimondi, Silvina Pinto, Valeria Olivera, Marina Dibárbora, Mariano Pérez-Filgueira, María Isabel Craig, Ariel Pereda

**Affiliations:** Laboratorio de Aves y Porcinos, Instituto de Virología CICVyA, Instituto Nacional de Tecnología Agropecuaria (INTA), CC25, B1712WAA Castelar, Buenos Aires Argentina; Cátedra de Patología Aviar, Facultad de Ciencias Veterinarias, Universidad de Buenos Aires (UBA), Chorroarín 280, C1427CWO CABA, Argentina; Consejo Nacional de Investigaciones Científicas y Tecnológicas (CONICET), Rivadavia 1917, C1033AAJ CABA, Argentina

## Abstract

Infection of poultry with chicken anemia virus (CAV) is implicated in several field problems in broiler flocks due to the immunosuppression generated and, consequently, the increased susceptibility to secondary infections. Recently, we have reported an increased occurrence of clinical cases caused by CAV strains distantly related to those commonly used for vaccination. In order to understand the behavior of two Argentinean CAV strains (CAV-10 and CAV-18) in two-week-old chickens, an immune and histopathological study was performed. Neither mortality nor clinical signs were observed in the infected or control groups. Thymus lobes from chickens infected with both CAV viruses were smaller compared to the negative control group. At 14 days post-infection (dpi), only chickens inoculated with CAV-10 show a severe depletion of lymphocytes in the thymus cortex and in follicles from the bursa of Fabricius. Also thymopoiesis disorders, such as reduction in the percentage of total DP (CD4 + CD8α+) thymocytes and alteration in the percentages of DP subpopulations, were more important in animals inoculated with the CAV-10 than the CAV-18 strain. In addition, only animals infected with CAV-10 show a decrease in CD8αβ splenocytes. Altogether our results show that, although both Argentinean CAV strains produce subclinical infections in chickens causing immunosuppression at 14 dpi, they might differ in their in vivo pathogenicity.

## Introduction

Chicken anemia virus (CAV) is a closed circular negative single-stranded DNA virus [1] from the *Circoviridae* family that belongs to the *Gyrovirus* genus [2]. The virus genome encodes a polycistronic mRNA from which three viral proteins are translated [3,4]: ORF1 encodes VP1, the only capsid protein; ORF2 encodes VP2, a scaffold protein which allows the proper folding of VP1 [5,6]; and ORF3 encodes VP3, which is known as an apoptin that induces apoptosis of thymocytes after in vivo infection and apoptosis of transformed avian cell lines after in vitro infection [7,8].

Worldwide, CAV causes considerable economic losses in the poultry industry because it is responsible for a clinical disease with high mortality characterized by anemia, haemorrhages and atrophy of the thymus and bone marrow in young chicks [9]. Infection of chickens older than two weeks of age, although considered subclinical, also has immunosuppressive effects with important consequences on growth and profitability of poultry [10–12]. Markowsky-Grimsrud and Schat [13] have demonstrated that experimental infection with CAV also impairs the generation of pathogen-specific cytotoxic T lymphocytes (CTL) that may have important implications for adaptive cell-mediated immunity to several secondary pathogens. This was also observed by other authors that reported secondary infections with other viruses, bacteria, parasites and/or fungi [1,14–16].

Experimental infection of chickens with CAV induced a substantial but transient decrease in some immunological parameters corresponding to a generalized lymphocytopenia in the thymus, bursa and spleen [17–19], with a peak at 14 days post-inoculation (dpi) and complete recovery at 28 dpi. These observations were observed in 1 or 14-day-old inoculated chickens [19,20]. While there is a consensus on the idea that absolute numbers of thymic lymphocytes dramatically decrease during the period of transient leukopenia [19–21], the selective alterations in the percentages of T lymphocyte subpopulations in the spleen during the acute phase is still under discussion. Coud et al. [19] showed a greater depletion of CTL than T-helper lymphocytes (ThL) in the spleen at 14 dpi, while Hu et al. [20] showed no selective decrease of ThL or CTL by flow cytometric analysis of CD4+ and CD8+ subpopulations. On the contrary, Vaziry et al. [21] showed a reduction in the percentage of CD4+ splenocytes only at 7 and 28 dpv, with a simultaneous increase in the percentages of CD8+ cells. It is noteworthy that most experimental infections with CAV have been done with reference strains such as CIA-1 or Cux-1 [19–24]; therefore, knowledge of the pathogenicity of CAV field strains remains poor and needs to be studied more.

In 2009, our group reported the circulation of CAV affecting both broilers and layers in commercial flocks in Argentina [25]. Sequence analysis of predicted VP1 peptides showed that most of the Argentinean viruses have a glutamine residue at positions 139 and 144, suggesting that these viruses might have a reduced spread in an avian lymphoblastoid cell line (MDCC-MSB1) compared with Cux-1 [26]. We showed that the circulation of CAV in commercial poultry in Argentina clearly affect their immune status through a subclinical infection [25]. Therefore, it would be important to broaden and deepen our knowledge about the effect of subclinical experimental infections with Argentinean CAV strains in chickens, moreover considering the high frequency of clinical cases and despite the fact that vaccination against CAV is common in breeder flocks in Argentina.

The present study addresses the effect of subclinical infection with two Argentinean CAV strains at 14 dpi. We studied the percentage of T-lymphocyte subpopulations in the thymus and spleen, and the histopathological changes produced in the thymus and bursa of Fabricius by both viruses. In addition, this paper contains the first two complete genome sequences of CAV published from Argentinean field strains and the first report of the behaviour of these viruses in vivo.

## Materials and methods

### Amplification and sequencing of the CAV genome

Firstly, a pair of primers was designed to obtain the complete genome of CAV (Table 1): CAV-NheI Fw and CAV-NheI Rv. The primers were used under cycling conditions of denaturation (5 min 95 °C), followed by 35 cycles of denaturation (30 s 95 °C), annealing (30 s 54 °C) and extension (5 min 72 °C). After a final extension (10 min 72 °C), samples were held at 16 °C, until visualization in a 1% agarose gel with ethidium bromide.

**Table 1:** **Sequences of the primers used to obtain the complete genome sequence of CAV strains**

**Oligo name**	**Sequence**	**Length**	**Position** ^**a**^
CAV-NheI Fw	5′-GCTAGCGTCAATGAACCTGA-3′	20-mer	1191-1210
CAV-NheI Rv	5′-GCTAGCAGGAACTCTTTCA-3′	19-mer	1178-1196
CAV-Genome Fw	5′-ACGCTAAGATCTGCAACT-3′	18-mer	651-668
CAV-Genome Rv	5′-GTAATTCCAGCGATACCAA-3′	19-mer	589-607
CAV-VP2 Fw	5′-ATGCACGGGAACGGCGGACAACC-3′	23-mer	380-402
CAV-VP3 Rv	5′- TTACAGTCTTATACACCTTCTTGCGGTTCG-3′	30-mer	822-851
CAV-Sec1 Rv	5′-AACGTCACTTTCGCAACGT-3′	19-mer	278-296
CAV-Sec4 Fw	5′-AGGGCAACGTTCAGTTTCTA-3′	20-mer	1833-1852

^a^GenBank accession no. M55918.

PCR products were purified with a QIAquick PCR purification kit (Qiagen) and sequencing was performed using the BigDye Terminator v3.1 Cycle Sequencing Kit on an ABI PRISM 3700 DNA Analyzer (Applied Biosystems) according to the manufacturer’s instructions. Sequences were carried out with the same amplification primers and internal primers CAV1, CAV2, CAV-VP1-A fw, CAV-VP1-A rev, CAV-VP1-B fw and CAV-VP1-B rev previously described [25,27]. Six new additional internal primers were designed to obtain the complete CAV genome through overlapping fragments: CAV-Genome Fw, CAV-Genome Rv, CAV-VP2 Fw, CAV-VP3 Rv, CAV-Sec1 Rv and CAV-Sec4 Fw (Table 1).

The consensus amino acid and nucleotide sequences for both CAV genomes were generated using Megalign (DNASTAR, Madison, WI, USA). Sequences were assembled and edited with Lasergene 8.1 (DNASTAR); BioEdit 7 was used for alignment and residue analysis.

### Chickens and housing

Specific-pathogen-free (SPF) White Leghorn embryonated eggs free of CAV antibodies were purchased (Rosenbusch S.A. CABA, Argentina), incubated and hatched in an automatic incubator (Yonar, CABA, Argentina) under appropriate conditions, and randomly separated into three groups of 6 chickens. Groups were housed separately in sterilized isolators for chickens under negative pressure conditions (Allentown CH8ISOL) with food and water *ad libitum* throughout the experimental period. Animal care and experimental procedures were performed in accordance with the approved protocols of the National Institute of Agricultural Technology Ethics Committee (INTA, Argentina).

### Viruses and inocula

The Argentinean CAV-10 and CAV-18 viruses were obtained from commercial broilers in 2007 with increase of mortality, gangrenous dermatitis and thymus and bursa atrophy [25]. These strains were unable to be grown in MDCC-MSB1 cells. Therefore, under our experimental conditions, inocula were adjusted to 10^6^ copies of CAV DNA per animal for comparative analyses of our results. Argentinean CAV viruses were propagated in chickens and CAV inocula were prepared from thymus samples as previously described [28]. Next, DNA was obtained with the QIAamp DNA Mini Kit (Qiagen Inc.Valencia, CA, USA) according to the manufacturer’s instructions, and the number of CAV genomes contained in each inoculum was determined by SYBR Green-based real-time quantitative PCR (RT-qPCR). Values were converted to number of copies of CAV DNA per μL of inoculum. Finally, the inocula were adjusted to 1 × 10^6^ copies of CAV DNA/200 μL.

### Quantitative Real Time PCR

SYBR Green-based quantitative real-time PCR (RT-qPCR) was used to determine the number of copies of the CAV genome in inocula.

#### Reagents

CAV DNA was amplified using reagents and primers purchased from Biodynamics. (CABA, Argentina). Primers used amplify a fragment (75 bp) located within the overlapping region between ORF2 and ORF3 (Table 2). The primers were designed and evaluated using Primer Express v1.0 software. Individual PCR reactions, containing 15 pmol of each primer in a total volume of 25 μL, were performed in an ABI Prism 7500 SDS system (Applied Biosystems, Warrington, UK).

**Table 2:** **Primers used in quantitative real time PCR to detect and quantify chicken anemia virus DNA**

**Primer**	**Sequence**	**Length**	**Position** ^**a**^
CAV-Real Time Fw	5′-AGAGAGATCCGGATTGGTATCG-3′	22-mer	576-597
CAV-Real Time Rv	5′-TGGGAGCGCGAGCATT-3′	16-mer	636-651

^a^GenBank accession no. M55918.

#### Cycling parameters

The cycling parameters consisted of the following steps: 95 °C hold for 10 min, followed by 40 cycles consisting of denaturation at 95 °C for 15 s and annealing/extension at 50 °C for 1 min, and finally adding a dissociation melting curve analysis.

#### Preparation of standards

A fragment of 75 bp of the CAV genome from Cux-1 vaccine strain was amplified by PCR using the primers described in Table 2 and cloned in pGEM T-Easy vector (Promega Madison, USA) to generate standard curves for the quantitation of number of copies of CAV DNA. Fresh tenfold dilutions were made from the plasmid stocks in distilled water for each PCR run to avoid the problem of plasmid degradation over time in the higher dilutions. Cycle threshold (CT) values were used to plot a standard curve and a range from 10^1^ to 10^6^ copy numbers was represented in each standard curve. The copy number was determined using the conversion factor: 1 ug of a 1-kb DNA = 3.03 pmol ends [29].

#### Analysis

The results from the PCR experiments were analyzed using Sequence Detection Systems v.1.6.3 software. The default settings of the program were used to define both the threshold value and baseline parameters for analysis of the raw data. A standard curve was generated in each assay and used to extrapolate the amounts in the unknown samples.

### Experimental design

Six two-week-old chickens were intramuscularly inoculated with 200 μL of CAV-10 or CAV-18 containing 1 × 10^6^ CAV DNA copies. Negative control group (*n* = 6) was inoculated with 200 μL of phosphate-buffered-saline (PBS). Signs of anorexia, weakness, stunting, cyanosis, petechiae and ecchymoses were looked for everyday in every bird. Fourteen days after inoculation, chickens were bled, euthanized and body, thymus, spleen and bursa of Fabricius were weighed and recorded in all birds. From each animal, the bursa and the first right lobe of the thymus were separated for histopathological evaluation. Fractions of the thymus and spleen were also kept for flow cytometry evaluation.

### Histopathology

Samples of the thymus and bursa of Fabricius were collected and immersed into 10% neutral buffered formalin for fixation. Samples were dehydrated and embedded in paraffin wax in the usual manner, sectioned (4 μm thick) and stained with hematoxylin and eosin (HE).

### Lymphocyte isolation

Single cell suspensions from spleens were obtained by direct mechanical disruption in RPMI 1640 through a 40 μm mesh (Cell Strainer, BD). Thymuses were cut into very small pieces and mechanically disrupted by pressing with a syringe plunger, in RPMI 1640. Then, cellular suspensions were passed through a 40 μm mesh (Cell Strainer, BD). Mononuclear cells were isolated from both suspensions by centrifugation over Histopaque density gradient (1.077 g/mL; Sigma, St. Louis, MO, USA) at 400 × *g* for 30 min and room temperature. Cells were isolated from the interface, washed, and live cells were counted using trypan blue exclusion.

### Flow cytometry analysis

Cells were diluted in staining buffer (PBS 1×, 10% FBS, 0.1% Sodium Azide) and 1 × 10^6^ cells per well were transferred into 96 well-plates (V-shape), and washed twice with the same buffer. Staining was performed by resuspending the cellular pellet of each well with 100 μL of staining buffer including different combinations of antibodies, or as single-color stainings for compensation. Cells were incubated at 4 °C for 30 min and washed twice with staining buffer by centrifugation at 250 × *g* for 5 min.

Avian monoclonal antibodies (mAbs) (CD3-SPRD, CD4-FITC, CD8α-PE, CD8α-FITC and CD8β-PE) were purchased from Southern Biotech (Birmingham, AL, USA). Mononuclear cell suspensions from every thymus and spleen were stained with one and two mAb mix, respectively: CD3-SPRD, CD4-FITC and CD8α-PE in tube A (for thymocytes and splenocytes), and CD3-SPRD, CD8α-FITC and CD8β-PE in tube B (splenocytes). All antibodies were titrated in order to determine the optimal staining concentration of each one.

Positive cells were analyzed with a FACS Calibur flow cytometer (BD Biosciences, San Jose, CA, USA) and CellQuest software. Analysis was done on 20 000 events and discrete viable lymphoid cell populations were gated according to the forward/side scatter characteristics. Percentages of different lymphoid cell subpopulations in the thymus and spleen were determined by multiparametric analysis.

### Statistical analysis

Data were tested for normal distribution prior to analysis (Shapiro-Wilk test) using the Statistical Package for the Social Sciences for Windows (SPSS, version 15.0, Chicago, IL, USA). An ANOVA test was used, and a Tukey’s test was applied when differences were detected. Variables that did not fit the ANOVA’s assumptions were analyzed by Kruskall-Wallis and Bonferroni-Dunn post hoc test. *P*-values of *p* < 0.05 were considered statistically significant.

### Serology

Sera were tested by competitive enzyme-linked immunosorbent assay (ELISA) for specific antibodies against CAV according to the manufacturer’s recommendations (Idexx Laboratories, Inc., Westbrook, ME, USA) using 10-fold dilutions of each serum sample.

### Nucleotide sequence accession numbers

The nucleotide genome sequence from Argentinean CAV strains obtained in this study are available from GenBank under accession numbers KJ872513 (CAV-10) and KJ872514 (CAV-18).

## Results

### Characterization of CAV field strains used for experimental infection of chickens

The complete nucleotide genome sequence of CAV-10 and CAV-18 was determined using primers described in Table 1. Both Argentinean CAV genomes are 2298 nt long, and lacked a 21-nt region located within the non-coding, transcription-regulatory region of the genome [4,30] that is present in the Cux-1 isolate (M55918). A comparison between the two Argentinean CAV viruses shows 14 individual nucleotide sequence differences dispersed throughout the genome, with three being located in the non-coding region and the rest along ORF1. Nucleotide differences between VP1 from CAV-10 and CAV-18 resulted in only 2 amino acid changes, one Q294H and the other S370T. In addition, both viruses present a glutamine (Q) residue at positions 139 and 144 of VP1.

### Clinical signs

No mortalities occurred after CAV or PBS inoculation during the experiment. No clinical signs of disease and no differences in body weight were observed between CAV-inoculated and negative control groups.

### Macroscopic alterations

**Figure 1:**
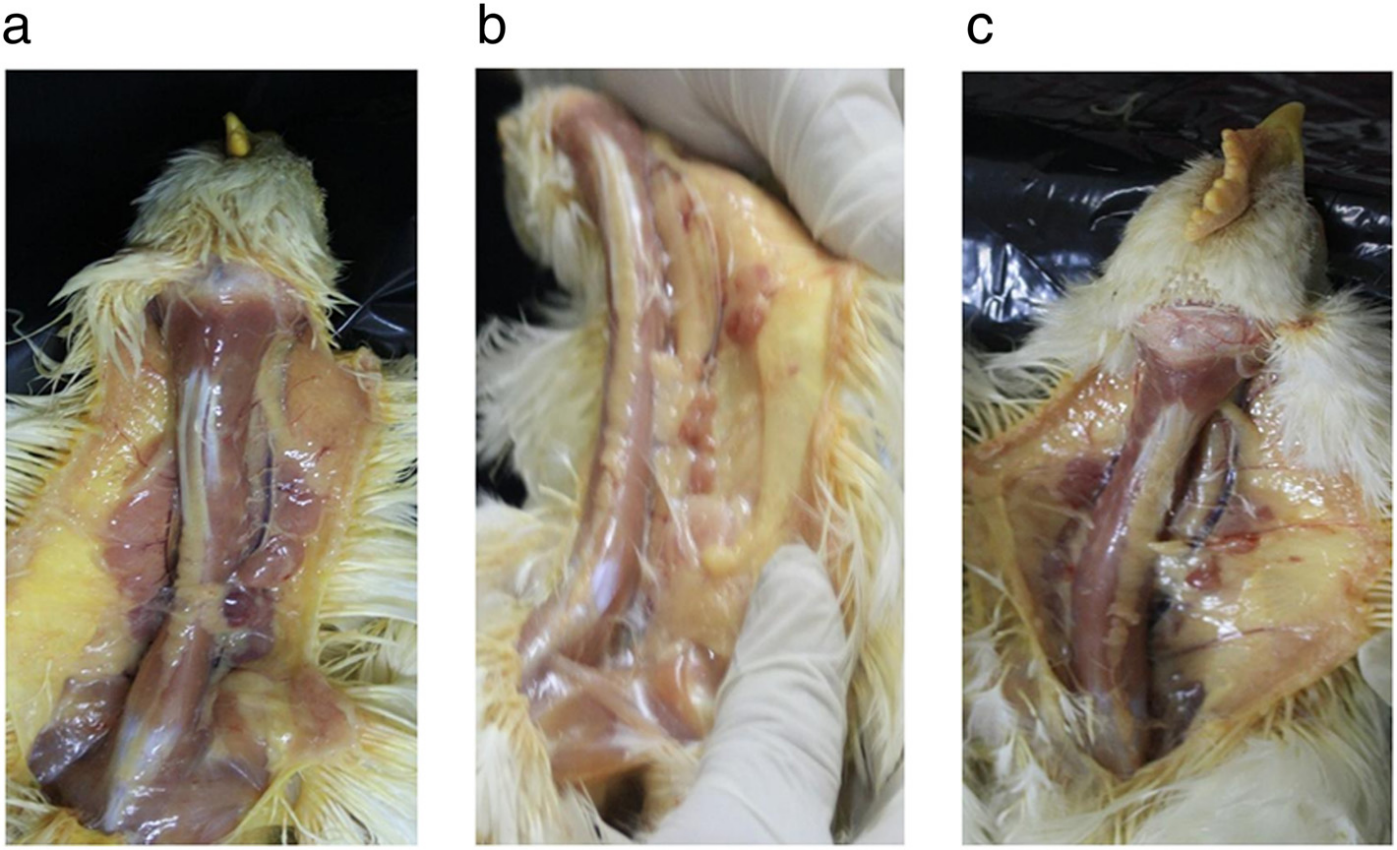
**Gross lesions after 14 days post-infection with Argentinean CAV strains in 2-week-old chickens.** Note that the thymic lobules from CAV-10 **(b)** and CAV-18 **(c)** inoculated chickens are smaller and reddened than those from the control group **(a)**.

Chickens inoculated with CAV-10 or CAV-18 show reddened and atrophied thymic lobes distributed on both sides along the neck. In contrast, the control group had whitish and firm thymic lobes (Figure 1). There were no other significant gross lesions in any of the groups.

**Figure 2:**
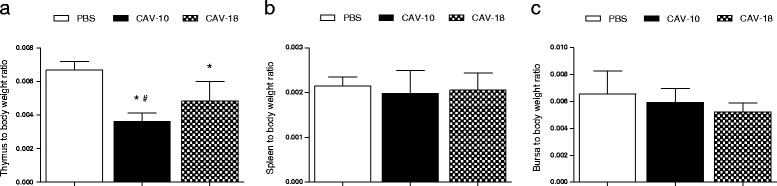
**Macroscopic alterations in chickens associated with chicken infectious anemia.** Alterations of thymus-to-body weight ratios **(a)**, spleen-to-body weight ratios **(b)** and bursa-to-body weight ratios **(c)** following infection with CAV-10 or CAV-18, 14 dpi. The results are expressed as the mean (SD) from each group, *n* = 6. *p < 0.05 vs. Control; # *p* < 0.05 vs. CAV-18.

Mean thymus-to-body weight ratios were reduced (*p* < 0.05) in both CAV-inoculated groups in comparison to the negative control group (Figure 2). In addition, these reductions were significantly different between CAV-10 and CAV-18 inoculated chickens. Spleens and bursas did not significantly alter their weight at this time post-infection.

**Figure 3:**
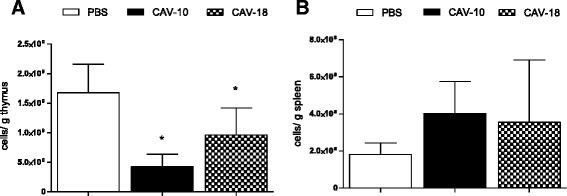
**Lymphocyte depletion after infection with Argentinean CAV strains.** Lymphocyte depletion expressed as the ratio of cells/g of thymus **(A)** or spleen **(B)** at 14 dpi in SPF chickens inoculated with CAV-10, CAV-18 or PBS at 2 weeks of age. The results are expressed as the mean (SD) from each group, *n* = 6. *p < 0.05 vs. Control.

Lymphocytes from mononuclear single cell suspensions from thymuses and spleens were counted using trypan blue exclusion, and cell depletion, expressed as the ratio between the number of cells per weight of the organ, was analyzed in both organs. A significant reduction of cell counts was observed in the thymuses from chickens inoculated with CAV strains as shown in Figure 3 (*p* < 0.05).

### Histopathological changes

**Figure 4:**
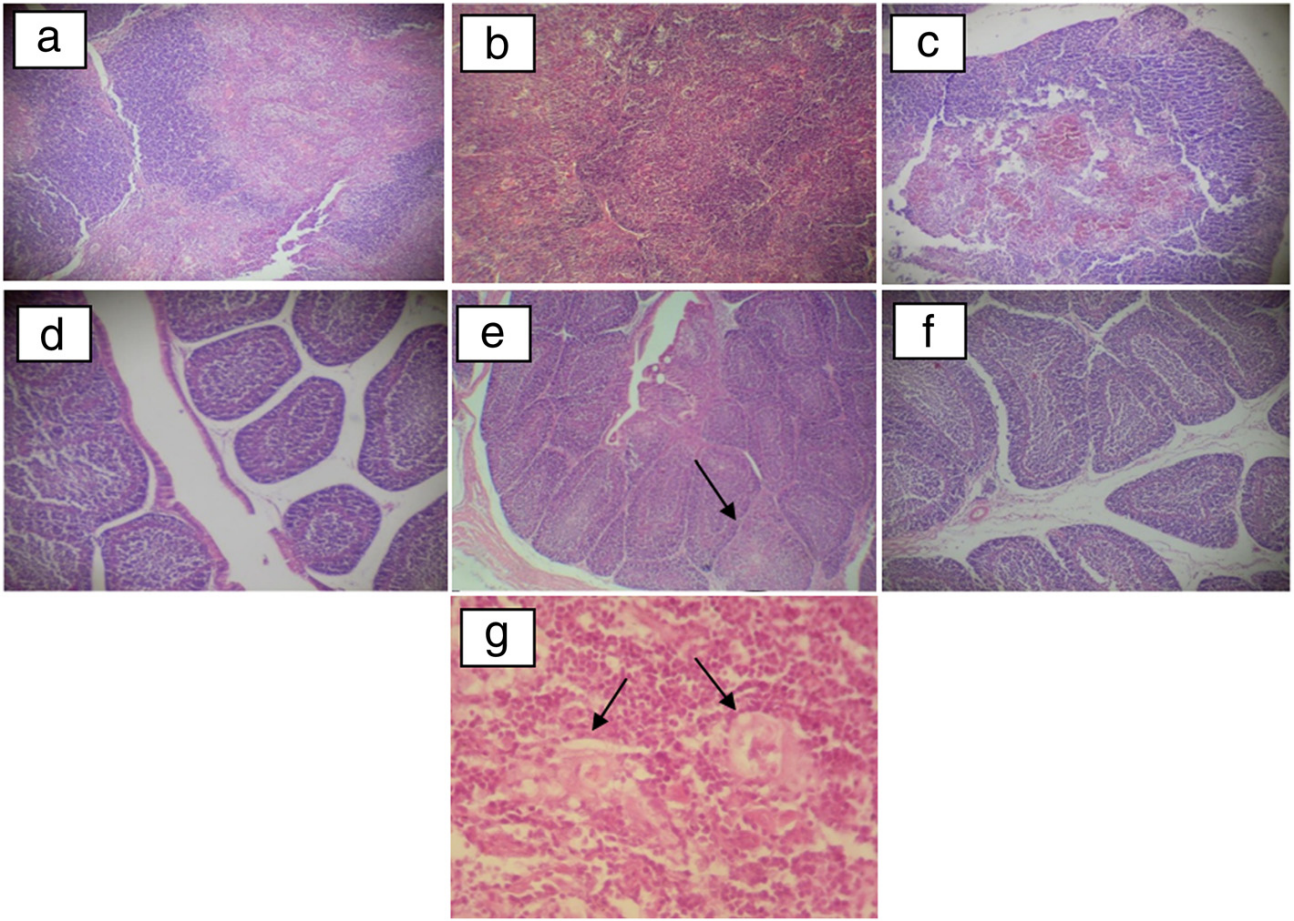
**Histopathological findings of CAV infection in tissues from 4-week-old SPF chickens.** Animals were studied 14 days after infection with CAV or inoculation with PBS at 2 weeks of age, and pictures show representative tissue samples from experimental and control groups stained with HE, 10× **(a-f). (a)** Thymus lobe from PBS inoculated group; **(b)** thymus lobe from CAV-10 inoculated group with severe cortical lymphocyte depletion and absence of demarcation between the medulla and cortex; **(c)** thymus lobe from CAV-18 inoculated group; **(d)** bursa of Fabricius from the PBS inoculated group; **(e)** bursa of Fabricius from CAV-10 inoculated group, showing moderate lymphoid depletion of the follicles with follicular atrophy marked with an arrow; **(f)** bursa of Fabricius from the CAV-18 inoculated group. **(g)** Large lymphoblasts with karyomegaly in thymuses from CAV-10 inoculated chickens, 40×; vacuolization of reticular cells were also marked by arrows.

On the one hand, thymuses from chickens inoculated with CAV-10 experienced severe lymphocyte depletion in the cortex (Figure 4b), showing a reduced demarcation between the medulla and cortex. Large lymphoblasts with karyomegaly and vacuolization of reticular cells were also observed in thymuses from CAV-10 inoculated chickens, producing a starry-sky appearance. Moreover, atrophy with moderate lymphoid depletion of the lymphoid follicles was observed in bursae from birds inoculated with CAV-10 (Figure 4e). Also, macrophage and plasma cell infiltration were observed in some of these bursae. On the other hand, thymus and bursa from CAV-18 inoculated chickens did not show histopathological changes at 14 dpi.

### Analysis of thymic cell subpopulations

**Figure 5:**
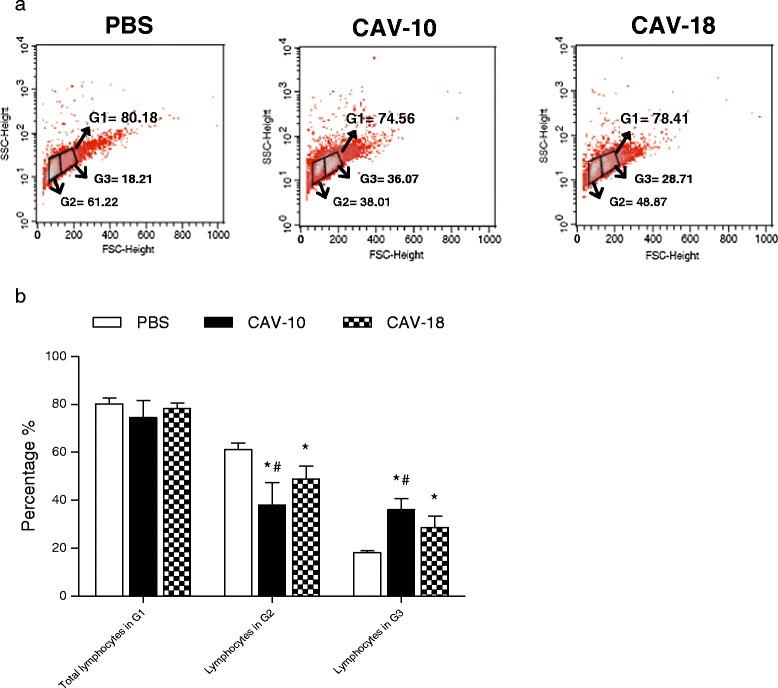
**Flow cytometry analysis of lymphocyte subpopulations in the thymus from CAV-10, CAV-18 and PBS inoculated chickens.** Animals were studied 14 days after infection with CAV or inoculation with PBS at 2 weeks of age. **(a)** Analysis was performed in G1 according to forward/side scatter parameters. Thymocytes in G1 were divided into small (G2) and large (G3) cells; **(b)** Mean percentages of G1, G2 and G3 cells in the thymus from control and CAV-inoculated groups. The results are expressed as the mean (SD) from each group, *n* = 6. *p < 0.05 vs. Control; # *p* < 0.05 vs. CAV-18.

To verify whether Argentinean CAV strains induce disorders in lymphoid cell populations or not, the percentages of different lymphoid subsets in thymuses from CAV-inoculated chickens were compared to those from control birds in gate 1 (G1) according to forward/side scatter parameters. Thymocytes in G1 were divided into small (G2) and large (G3) cells to verify if thymopoiesis was also altered by CAV infection (Figure 5a).

Although the comparison of the lymphocyte percentages from each group shows no significant differences in G1, percentages in G2 and G3 were significantly different between groups (Figure 5b). There was a clear and important reduction in the percentages of lymphocytes in G2 from chickens inoculated with Argentinean CAV viruses (*p* < 0.05). In addition, CAV-10 show a higher reduction of lymphocytes in G2 than CAV-18 (*p* < 0.05). In contrast, the percentages of lymphocytes in G3 were increased in both CAV inoculated groups (*p* < 0.05). This increase was higher in thymuses from CAV-10 inoculated chickens with respect to the increase observed in thymuses from CAV-18 inoculated birds (*p* < 0.05).

**Figure 6:**
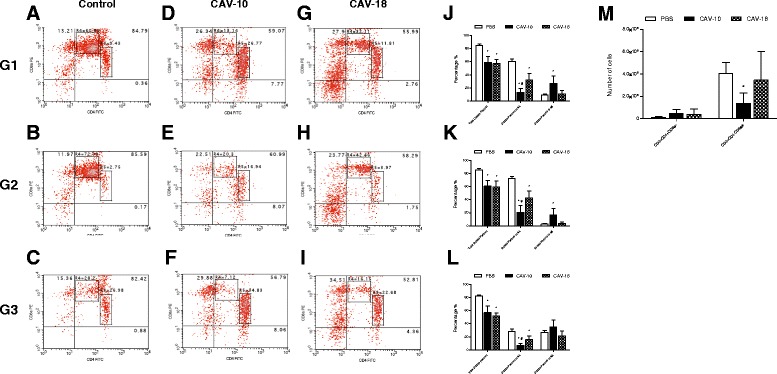
**Analysis of different lymphocyte subpopulations in the thymus of SPF chickens.** Animals were studied 14 days after infection with CAV or inoculation with PBS at 2 weeks of age. **(A-I)** CD4 + CD8α-, CD4-CD8α + and CD4 + CD8α + (DP) thymocytes from one thymus from each group in G1 **(A,** **D,** **G),** G2 **(B,** **E,** **H)** and G3 **(C,** **F,** **I)**. R4 and R5 represent DP thymocytes with different CD4 and CD8α surface expression. **(J-L)** Mean percentage of total DP, DP in R4 and DP in R5 thymocytes in G1, G2 and G3 from control (□), CAV-10 (■) and CAV-18 () birds. **(M)** Mean absolute number of CD4 + CD8α- and CD4-CD8α + thymocytes in G1 from control (□), CAV-10 (■) and CAV-18 () birds. Cells were triple-labeled with anti-CD3, anti-CD4 and anti-CD8α antibodies conjugated to SPRD, FITC and PE, respectively, and analyzed by flow cytometry. The results are expressed as the mean (SD) from each group, *n* = 6. *p < 0.05 vs. Control; # *p* < 0.05 vs. CAV-18.

The multiparametric analysis of CD3+ cells within the G1 region revealed the presence of CD4 + CD8α-, CD4-CD8α + and CD4 + CD8α + cell subsets. As shown in Figure 6A, the predominant subset in the control thymus was the double positive (DP) CD4 + CD8α+. Preliminary data show that these DP cells also express CD8β on their surface (data not shown), indicating that they are immature DP cells that did not undergo positive and negative selection, a characteristic during ontogeny of T lymphocytes. These DP cells in G1 were then divided into two regions for further analysis, R4 and R5, according to the surface expression of CD4 and CD8α. While thymocytes in R4 have higher CD8α expression than thymocytes in R5, thymocytes in R5 have higher CD4 expression than cells in R4.

Figures 6B and 6C show DP cells from a thymus of the control group in G2 and G3 respectively. Most of the small DP with size and granularity compatible with G2 were in R4, whereas large DP cells from G3 were located in both R4 and R5. In particular, CAV-10 not only reduced (*p* < 0.05) the percentages of total DP thymocytes in G1, G2 and G3 as CAV-18 (Figure 6J-L), but also altered the percentage of DP thymocytes in R4 and R5 (Figure 6D-I). CAV-10 in particular produced a higher reduction of DP in R4 (in G1, G2 and G3) than CAV-18 (*p* < 0.05), and a particular significant increase of DP in R5 (G1 and G2) that was not seen in thymuses from CAV-18 inoculated chickens (Figure 6J-L).

Figures 6A, 6D and 6G also show some differences in CD4 + CD8α- and CD4-CD8α + thymocytes in G1 between control, CAV-10 and CAV-18 inoculated groups. Both CAV-inoculated groups appear to have increased their CD4 + CD8α- and CD4-CD8α + subsets when relative percentages were analyzed; however, when the absolute number of CD4 + CD8α- and CD4-CD8α + thymocyte subpopulations were compared between groups, only the number of CD4-CD8α + thymocytes were reduced in thymuses from CAV-10 inoculated chickens (*p* < 0.05) (Figure 6M).

### Analysis of splenic cell subpopulations

Splenic lymphocytes from experimental groups were analyzed in G1 according to forward/side scatter parameters.

#### Staining with CD3/CD4/CD8α

**Figure 7:**
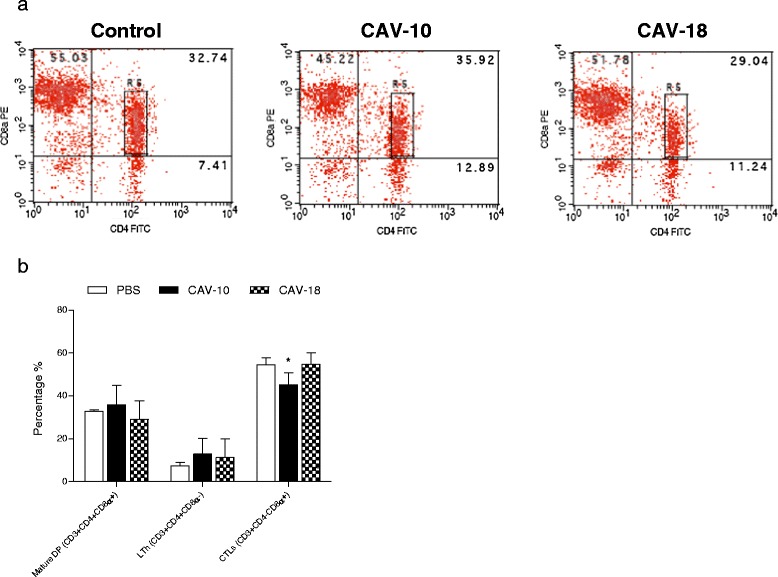
**Flow cytometry analysis of CD4 + CD8α-, CD4-CD8α + and CD4 + CD8α + (DP) splenocytes of SPF chickens. (a)** Analysis was performed in G1 according to forward/side scatter parameters. Most double positive splenocytes were in R5 according to their CD4 and CD8α surface expression. **(b)** Mean percentage of splenocyte subpopulations from control (□), CAV-10 (■) and CAV-18 () birds. Cells were triple-labelled with anti-CD3, anti-CD4 and anti-CD8α antibodies conjugated to SPRD, FITC and PE, respectively, and analysed by flow cytometry. The results are expressed as the mean (SD) from each group, *n* = 6. *p < 0.05 vs. Control.

The analysis revealed the presence of CD3 + CD4 + CD8α- (ThL), CD3 + CD4-CD8α + (CTL) and CD3 + CD4 + CD8α + (mature DP) cell subsets. Figure 7a shows that mature DP cells from spleen were in R5, indicating that there was a unique DP subset that represents a particular splenic population. These mature DP cells were not significantly altered at 14 dpi with Argentinean CAV strains (Figure 7b). Similar results were obtained for splenic ThL cells, which did not significantly differ between groups. However, the CTL subset decreased only in chickens inoculated with CAV-10 (*p* < 0.05).

#### Staining with CD3/CD8α/CD8β

**Figure 8:**
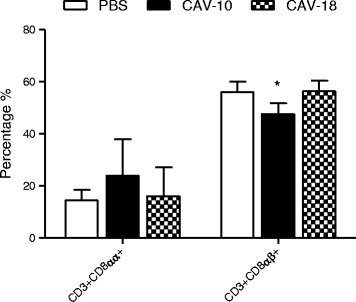
**Analysis of CD3 + CD8αα + and CD3 + CD8αβ + splenocytes of SPF chickens.** Analysis was performed in G1 according to forward/side scatter parameters. Splenocyte subpopulations from control (□), CAV-10 (■) and CAV-18 () birds were triple-labelled with anti-CD3, anti-CD8α and anti-CD8β antibodies conjugated to SPRD, FITC and PE, respectively, and analysed by flow cytometry. The results are expressed as the mean (SD) from each group, *n* = 6. *p < 0.05 vs. Control.

The G1 region revealed the presence of splenic CD8 lymphocytes, either CD8αα or CD8αβ cell subsets. The percentages of splenic CD8αα cells did not significantly differed between groups, although a small increase was observed in CAV-10 inoculated chickens (Figure 8). In contrast, the number of CTL CD8αβ was reduced in chickens inoculated with CAV-10 (*p* < 0.05).

### Serological testing

**Figure 9:**
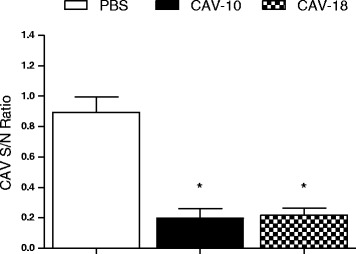
**Specific antibody levels against CAV after 14 days post-infection with Argentinean CAV strains in 2-week-old chickens.** Optical densities obtained were expressed as signal to noise ratios. The results are expressed as the mean (SD) from each group, *n* = 6. *p < 0.05 vs. Control.

Before inoculation, 3 chickens per group were bled, and all of them were negative to antibodies against CAV. At 14 dpi, seroconversion of animals inoculated with CAV-10 and CAV-18 was monitored. All chickens from CAV-10 and CAV-18 inoculated groups were positive for anti-CAV antibodies (S/N ratio ≤ 0.6), whereas birds from the control group remained negative (S/N ratio > 0.6). At this time post-infection no significant differences in CAV-specific antibody levels could be detected between CAV-10 and CAV-18 inoculated groups (Figure 9).

## Discussion

Complete genome sequencing and comparison of CAV-10 and CAV-18 viruses showed few differences between these two Argentinean strains. Both CAV strains have a deletion that contained a fifth 19-nt repeat, with this region being associated with a better efficiency to replicate in MDCC-MSB1 cell cultures [31]. However, previous reports affirmed that the contribution of this region to the attenuation or pathogenicity of CAV in chickens is still questionable [24].

In addition to the deletion, genome sequencing of CAV-10 and CAV-18 confirmed that both viruses present mutations in the hypervariable region of VP1 that are also associated with a reduced spread in an avian lymphoblastoid cell line (MDCC-MSB1) compared with Cux-1 [26]. However, these authors were unable to demonstrate differences in host pathogenesis in vivo due to differences in this region. On the contrary, Meehan et al. [24] have shown that this region does not contribute disproportionately to pathogenicity compared to other regions throughout the genome, and that alterations in more than one of the three CAV proteins, as well as in the noncoding region of the genome, may influence pathogenicity.

Despite the eleven nucleotide differences observed along VP1, only two deduced amino acid substitutions were seen in CAV-10 with respect to the CAV-18 virus. To our knowledge, there are still no reports about the plausible role or the way in which these differences may affect the pathogenesis of CAV in the chicken. We believe that this report could be the first approach to evaluate these amino acid differences of VP1 in vivo, moreover taking into account the few nucleotide variations between CAV-10 and CAV-18 observed only in the non-coding region. However, under our experimental conditions we cannot ensure that the same dosage of each isolate was administered in chickens. Therefore, further studies should be performed to calculate infectivity titres of these field strains in vitro to associate genomic differences between Argentinean CAV strains with different pathogenicities in vivo.

In the present study chickens infected either with CAV-10 or CAV-18 did not present clinical signs of disease throughout the experiment. However, both groups inoculated with Argentinean CAV strains experimented a significant reduction of thymus-to-body weight ratios (CAV-10 produced a significantly higher reduction than CAV-18) and thymocyte depletion at 14 dpi. These results suggest that both CAV field strains produce a subclinical infection in 2-week-old birds.

In addition, chickens infected with CAV-10 virus also show histopathological lesions in the thymus and bursa, compatible with those previously reported by Haridy et al. [32] in subclinical infections with CAV. It is important to mention that these authors infected animals intramuscularly using an isolate that was passaged 10 times in MDCC-MSB1 cells [33], where mutations could be introduced. By contrast, CAV-18 inoculated chickens did not present obvious morphological changes in the evaluated organs. This might suggest that alteration of functionality was not very strong or that their course was produced in a very short time in this group and, consequently, the morphological changes could not be observed throughout a histopathological study. Therefore, differences in histopathological lesions produced by CAV-10 or CAV-18 in 2-week-old chickens might suggest that CAV-10 is more aggressive than CAV-18 after in vivo infection, at least at this time post-infection.

Flow cytometry analysis of thymocytes in G1, G2 and G3 from CAV-inoculated chickens vs. control group animals suggests that Argentinean CAV strains alter the thymocyte maturation process after 14 dpi.

The most evident variation was the reduction observed in DP thymocytes following CAV inoculation. Similar results were published by Vaziry et al. [21] and Hu et al. [20], evaluating the lesions in 1-day-old chicks infected with the CIA-1 strain. In addition, our results clearly demonstrate that there are different stages of maturation of DP cells due to thymopoiesis in the thymus, and that some of these stages could be altered after CAV infection. While DP in R4 significantly decreased after CAV infection, DP in R5 significantly increased. These evident changes in the percentages of DP thymocytes with different CD4 and CD8α surface expression support the idea that Argentinean CAV viruses can alter thymopoiesis. Even more, under our experimental conditions, CAV-10 produced significantly deeper alterations than CAV-18 in thymocyte maturation two weeks after infection.

On the contrary, the reduction in the total number of lymphocytes in thymuses from CAV inoculated chickens observed during the cell isolation procedure, the absence of significant changes in the number of CD4 + CD8α- thymocytes and the slight decrease of CD4-CD8α + cells observed after flow cytometry analysis of the thymic population add to the notion that mature lymphocytes were not recruited into the thymus at 14 dpi with Argentinean CAV strains. These results were in concordance with previous observations by Vaziry et al. [21] in a subclinical infection with a vaccinal strain.

Finally, the alterations in percentages of splenic lymphocyte subpopulations were also analyzed by flow cytometry. There were no differences in mature DP and ThL cells between groups after infection with Argentinean field strains, similar to results previously published by other authors with the CIA-1 vaccinal strain [21]. However, in our experimental infection, CTL cells were statistically reduced in spleens from CAV-10 inoculated chickens. Moreover, staining with CD3, CD8α and CD8β mAb allowed discrimination between CD8+ subsets (CD8αα or CD8αβ) that were affected by CAV inoculation. While our results show no differences in CD8αα cells, significant reduction in percentages of the CD8αβ subpopulation was observed only in CAV-10 inoculated birds. Adair et al. [22] have demonstrated the presence of infected mature lymphocytes in the spleen after 6 dpi, although the numbers of infected cells as a proportion of the total number recorded was much lower than that in the thymus or bone marrow. Our results, where a significant decrease in splenic CD8αβ lymphocytes was observed at 14 dpi, could be the consequence of the large amount of CAV-infected splenocytes reported by Adair et al. at a previous time post-infection. Altogether, our results suggest that CAV-10 affects splenic lymphocyte subpopulations by a reduction in CD8αβ subset and, consequently, this could alter the host immune response against other secondary pathogens.

In this work we studied the subclinical infection of 2-week-old chickens with two Argentinean CAV field strains, showing some nucleotide differences in their genome sequence. Under our experimental conditions, the results might suggest there are differences in the behavior of CAV-10 and CAV-18 strains in vivo at 14 dpi. Further studies should be done to determine first the viral titer of these CAV field strains and then, to associate nucleotide/amino acidic composition, in VP1 and/or in non-coding region, with differential pathogenicity in chickens.

## List of abbreviations

CAV: Chicken Anemia Virus
CAV-10: Chicken Anemia Virus, Argentinean strain 10
CAV-18: Chicken Anemia Virus, Argentinean strain 18
CT: Cycle threshold
DP: Double Positive
dpi: Days post-infection
ELISA: Enzyme-Linked ImmunoSorbent Assay
HE: Hematoxylin and eosin stain
MDCC-MSB1: Chicken hematopoietic lymphoblast cell line
ORF: Open Reading Frame
PCR: Polymerase Chain Reaction
RT-qPCR: Real-time quantitative PCR
